# Combustion-derived particles inhibit in vitro human lung fibroblast-mediated matrix remodeling

**DOI:** 10.1186/s12951-018-0410-x

**Published:** 2018-10-27

**Authors:** Hannelore Bové, Jens Devoght, Leentje Rasking, Martijn Peters, Eli Slenders, Maarten Roeffaers, Alvaro Jorge-Peñas, Hans Van Oosterwyck, Marcel Ameloot

**Affiliations:** 1 Biomedical Research Institute, Hasselt University, Agoralaan Building C, Diepenbeek, Belgium; 2 Centre for Surface Chemistry and Catalysis, KU Leuven, Celestijnenlaan 200F, Louvain, Belgium; 3 Institute for Materials Research, Hasselt University, Agoralaan Building D, Diepenbeek, Belgium; 4 Department of Mechanical Engineering, KU Leuven, Celestijnenlaan 300C, Box 2419, Louvain, Belgium; 5 Prometheus, div. Skeletal Tissue Engineering, KU Leuven, Louvain, Belgium

**Keywords:** Combustion-derived particles, In vitro toxicology, Human lung fibroblasts, Hazard assessment, Matrix remodeling inhibition

## Abstract

**Background:**

The continuously growing human exposure to combustion-derived particles (CDPs) drives in depth investigation of the involved complex toxicological mechanisms of those particles. The current study evaluated the hypothesis that CDPs could affect cell-induced remodeling of the extracellular matrix due to their underlying toxicological mechanisms. The effects of two ultrafine and one fine form of CDPs on human lung fibroblasts (MRC-5 cell line) were investigated, both in 2D cell culture and in 3D collagen type I hydrogels. A multi-parametric analysis was employed.

**Results:**

In vitro dynamic 3D analysis of collagen matrices showed that matrix displacement fields induced by human lung fibroblasts are disturbed when exposed to carbonaceous particles, resulting in inhibition of matrix remodeling. In depth analysis using general toxicological assays revealed that a plausible explanation comprises a cascade of numerous detrimental effects evoked by the carbon particles, including oxidative stress, mitochondrial damage and energy storage depletion. Also, ultrafine particles revealed stronger toxicological and inhibitory effects compared to their larger counterparts. The inhibitory effects can be almost fully restored when treating the impaired cells with antioxidants like vitamin C.

**Conclusions:**

The unraveled in vitro pathway, by which ultrafine particles alter the fibroblasts’ vital role of matrix remodeling, extends our knowledge about the contribution of these biologically active particles in impaired lung tissue repair mechanisms, and development and exacerbation of chronic lung diseases. The new insights may even pave the way to precautionary actions. The results provide justification for toxicological assessments to include mechanism-linked assays besides the traditional in vitro toxicological screening assays.

**Electronic supplementary material:**

The online version of this article (10.1186/s12951-018-0410-x) contains supplementary material, which is available to authorized users.

## Background

Human exposure to combustion-derived carbonaceous particles (CDPs) has increased substantially in recent years. Traffic exhaust, for example, constitutes a major environmental contaminant of diesel soot or black carbon (BC) derived from the incomplete combustion of fuels. Additionally, multiple carbon black (CB) particle types are intentionally engineered via controlled combustion processes for their use in consumer products like printer toner cartridges, car tires and cosmetics. Consequently, hazardous exposure to this type of particles is not limited to occupational settings but also includes daily receptivity of environmental pollutant particulates [1]. While carbon black and black carbon are different particle types, both are classified as CDPs and find commonality in their combustion-based production process, resembling physicochemistry and demonstrated toxicity in various models [1].

The respiratory system is a major route of unintentional exposure to aerosolized carbonaceous particles. Once inhaled, CDPs can reach the deepest regions of the respiratory tract depending on their sizes. Especially, fine (diameter < 2.5 μm) and ultrafine particles (diameter < 0.1 μm) tend to deposit in the deeper bronchial-alveolar regions of the lungs where they are not rapidly degraded but accumulate and, eventually, may translocate to the blood and other target organs [1–4].

There is increasing evidence that exposure to CDPs can lead to numerous adverse health effects [1]. Moreover, these particles are thought to be more harmful to human health than particulate matter generated by other means [5–7]. Grahame and Schlesinger, for instance, have concluded that BC particles are the dominant environmental cause of cardiovascular morbidity and mortality [8]. Furthermore, epidemiological and experimental studies have shown that CDPs may attribute to the modulation and aggravation of pulmonary disorders and can even lead to lung cancer [1, 9, 10]. The constant human exposure to CDPs warrants in depth investigation of the involved toxicological mechanisms of these particles inducing the observed illnesses. Both in vitro and in vivo studies have already elucidated that carbonaceous particle exposure can induce cytotoxic injury, impaired redox regulation, inflammation and tissue remodeling [1, 11, 12]. In chronic lung diseases, to which inhalation of CDPs appears to contribute, tissue remodeling has shown to contribute to structural and functional alterations in the lungs. Yet, the exact toxicological mechanisms involved and their interconnections are still not fully unraveled. In a previous study by our research groups, we have shown that the tubulin cytoskeleton of fibroblasts is heavily disturbed after carbonaceous particle exposure [13]. Consequently, we hypothesize that CDPs may impair cell-induced remodeling of the extracellular matrix (ECM) due to their underlying toxicological mechanism.

Hence, the current study was designed to quantitatively evaluate whether CDPs affect the cellular remodeling of the surrounding matrix and to decipher the distinct toxicological mechanism underlying this detrimental effect. The study was performed using lung fibroblasts. Despite the fact that lung epithelial cells and macrophages constitute the first barrier in the lungs, fibroblasts are as critical to evaluate since they are the main connective tissue cell type and maintain the stroma for numerous other cells including alveolar epithelial cells [14]. In damaged lung tissue, fibroblasts are responsible for the main repair mechanisms including building new ECM and contracting the novel matrix to match it with undamaged tissue [14, 15]. Consequently, it is of critical importance to evaluate their remodeling capacity under CDP exposure. Here, 3D cell-induced displacement microscopy (CDM) is employed to examine the detrimental effects of CDPs on human lung fibroblasts.

## Results

### Cytoskeletal integrity

The effects of one fine (CCB; diameter < 2.5 µm) and two ultrafine (ufPL and ufP90; diameters < 0.1 µm) CDP types were tested of which all key physicochemical characteristics are summarized in Additional file 1: Table S1.

When studying the actin cytoskeleton, it can be seen that the filaments are disturbed after an incubation period exceeding 4 h (Additional file 1: Figure S1). Moreover, no clear stress fibers are observable compared to the control cell. In agreement with our previous report about the disturbance of the tubulin cytoskeleton after CDP exposure [13], the larger, fine CCB particles have a less pronounced effect on the cytoskeleton than the smaller ultrafine ufPL and ufP90 particles (data not shown).

### 3D cell-induced displacements and matrix remodeling

**Fig. 1:**
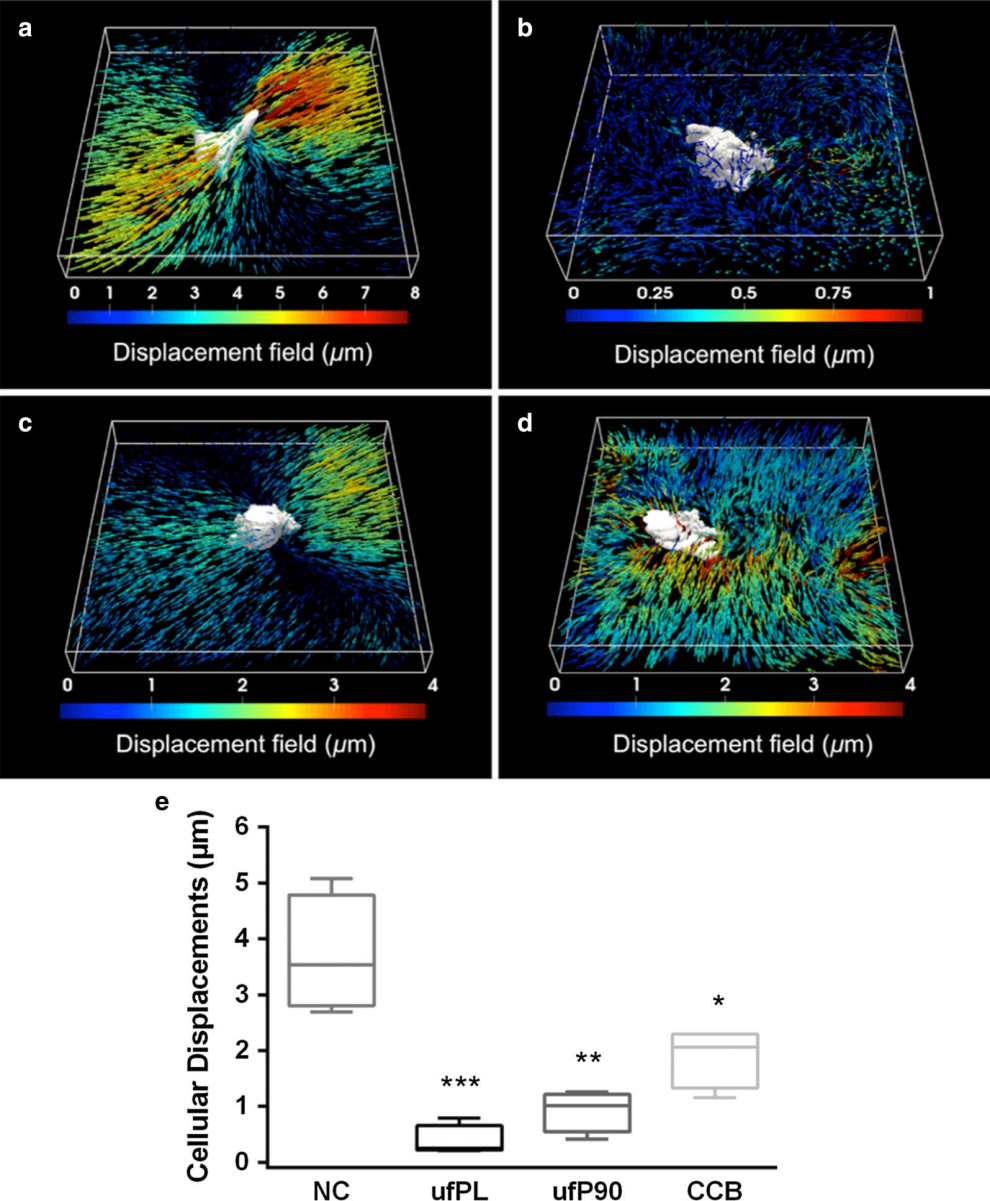
Impaired human lung fibroblast displacements in 3D collagen type I hydrogels induced by CDPs. Human lung fibroblasts (MRC-5 cell line) were exposed for 24 h to 20 µg/cm^2^ of three different types of CDPs at 37 °C and embedded in 3D collagen type I hydrogels to study their ability to generate matrix displacements. Representative 3D displacements induced by fibroblasts (cell body in white) incubated with **a** culture medium (NC, negative control), bounding box 172 × 172 × 21 µm^3^; **b** ufPL, bounding box 173 × 172 × 46 µm^3^; **c** ufP90, bounding box 167 × 170 × 11 µm^3^; and **d** CCB, bounding box 172 × 172 × 26 µm^3^; obtained from the registration of fibril-based images. Note, on the right some displacements of a lower localized cell are visible. **e** Data analysis of the 3D distribution of matrix displacements under the different conditions. Data are represented as box-plots (medians and quartiles; n = 4). Statistically different from control *p < 0.005; **p < 0.0005; ***p < 0.0001

Representative images showing 3D collagen matrix displacements induced by embedded fibroblasts exposed to plain culture medium (negative control) or three different CDP types are shown in Fig. 1a–d. The images indicate that the displacements induced by control cells are significantly different from cells exposed to ultrafine and fine particles. This visual observation is confirmed by quantitative analysis (Fig. 1e). The median ± standard deviation (SD) of 3D cell-induced matrix displacements drops from 4 ± 1 µm (negative control) to 0.4 ± 0.3, 0.9 ± 0.4 and 2 ± 0.5 µm for the ultrafine (ufPL, ufP90) and fine (CCB) particles, respectively. Also, it is clear that the ultrafine particles are causing more detrimental effects to the cells ability of structuring the ECM than the larger particles.

**Fig. 2:**
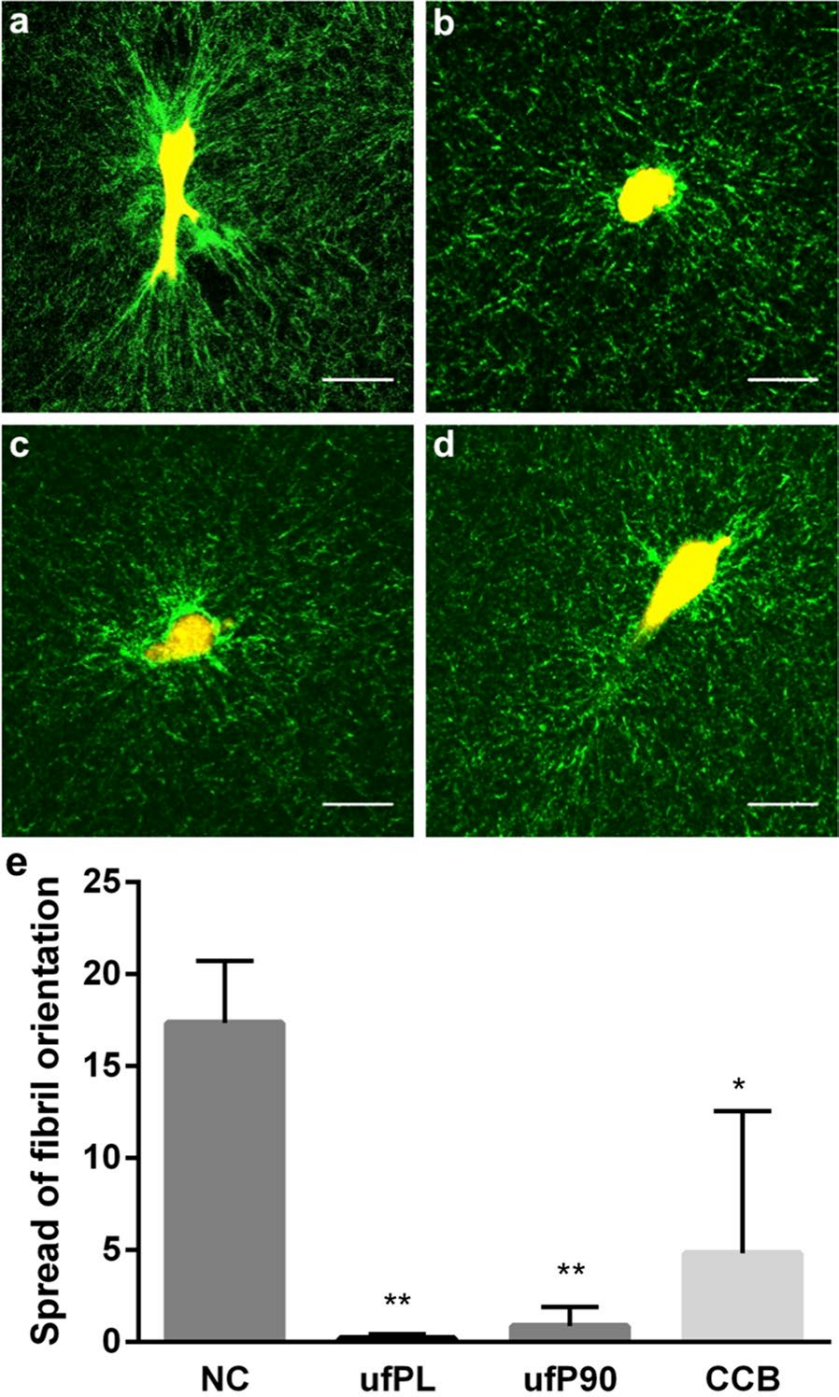
Inhibited collagen type I matrix remodeling by human lung fibroblasts exposed to CDPs. Human lung fibroblasts (MRC-5 cell line) were exposed for 24 h to 20 µg/cm^2^ of three different types of CDPs at 37 °C and embedded in 3D collagen type I hydrogels to study cell-mediated matrix remodeling. Representative images of collagen type I (second harmonic imaging, green) remodeling induced by fibroblasts (cell body in yellow) are shown incubated with **a** culture medium (negative control), **b** ufPL, **c** ufP90, and **d** CCB. Scale bars: 30 µm. **e** Analysis of the spread of fibril orientation of the collagen fibrils (*κ*) as quantification of the collagen organization around the force poles of the embedded cells under the various conditions. Data are represented as means ± standard deviation (SD) (n = 4). Statistically different from control *p < 0.005; **p < 0.0005

The findings correspond well with changes observed in the organization of the ECM after fibroblast remodeling as can be seen from the label-free imaging of the fibrils of the collagen type I matrix using second harmonic generation (Fig. 2a–d). Whereas the control cells nicely contract and align the collagen fibrils at their force poles, the fibril distribution is more randomly oriented around cells exposed to CDPs. The matrix distribution was quantified based on fibril orientation histograms with fitted Von Mises function (Additional file 1: Figure S2). The median ± SD spread of fibril orientation (*κ*) varies from 17 ± 3 (negative control) to 0.2 ± 0.2, 0.9 ± 1.0 and 5 ± 8 for the ultrafine (ufPL, ufP90) and fine (CCB) particles, respectively Fig. 2e). The lower values after incubation with fine but especially ultrafine particulates indicate that the collagen fibrils are randomly oriented compared to the control condition where the fibrils are highly aligned around the cellular force poles.

To examine if inhibition of cell-mediated matrix remodeling results from cytotoxicity induced by the particles, the extent and mode of cell death was investigated. Analysis showed that ultrafine but not fine carbonaceous particles at a concentration of 20 µg/cm^2^ are able to induce significant apoptotic cell death in human lung fibroblasts (Additional file 1: Figure S3).

### ROS generation

To investigate the possibility of a ROS-mediated mechanism responsible for the inhibition of the cellular displacements and resulting impaired matrix remodeling, ROS assays under abiotic (absence of cells) and biotic (presence of cells) conditions were executed.

**Fig. 3:**
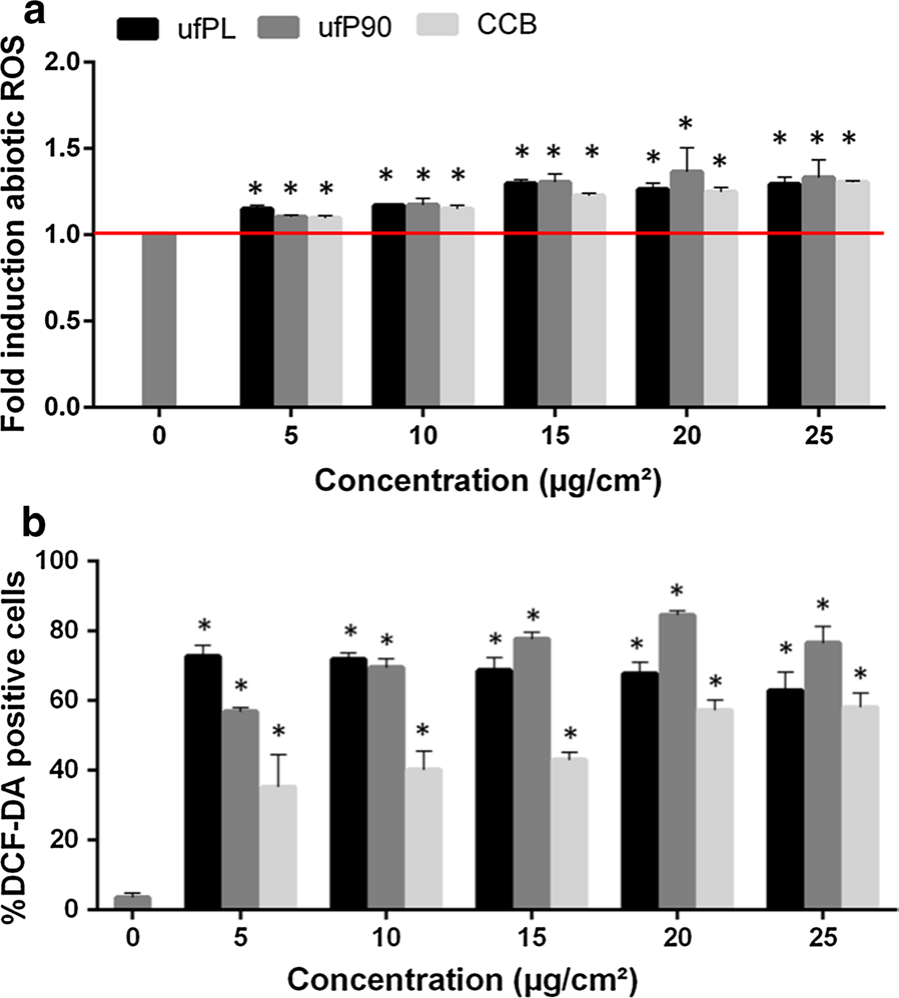
ROS production of CDPs under abiotic and biotic conditions. **a** Inherent oxidative potential of the three different CDPs at the various concentrations tested in this study. Red line: the background oxidative level in blank cell culture (IMDM) solution was set as reference. Data are represented as means ± SD (n = 3). * Statistically different from control p < 0.05. **b** Human lung fibroblasts (MRC-5 cell line) were exposed to different concentrations (5–25 µg/cm^2^) of three different types of CBs for 24 h at 37 °C. At the end of the exposure a DCF-DA assay was conducted to determine ROS production under biotic conditions. All data are represented as means ± SD (n = 3). * Statistically different from control p < 0.0001

The inherent oxidative ability of the different CDPs in abiotic conditions was measured using a DTT assay. All CDPs oxidized DTT in a dose-dependent manner (Fig. 3a). In addition, the abiotic ROS production for all concentrations was significantly different from the control condition.

To further explore the role of oxidative stress under biotic conditions, a DCF-DA staining was performed. In the presence of a wide variety of ROS, the non-fluorescent cell-permeant dye is converted to highly fluorescent cell-retained species. The percentage of fluorescent-positive cells was used for assessing the extent of ROS production in human lung fibroblasts. The analysis showed significant increase in ROS production in cells after 24 h incubation at even the lowest CB concentration (Fig. 3b). Moreover, at least eightfold ROS generation compared to the control is detected. No clear dose-dependent response curves are observed for the ultrafine particles, in contrast the ROS production stagnates or even declines at higher concentrations.

Contradictory to our results under abiotic conditions, the intracellular ROS production significantly varies between CDP types (p < 0.05). Also, the ROS analysis elucidated similar results for both types of ultrafine particles, while the larger, fine particles showed less ROS production.

### Mitochondrial organization and functioning

**Fig. 4:**
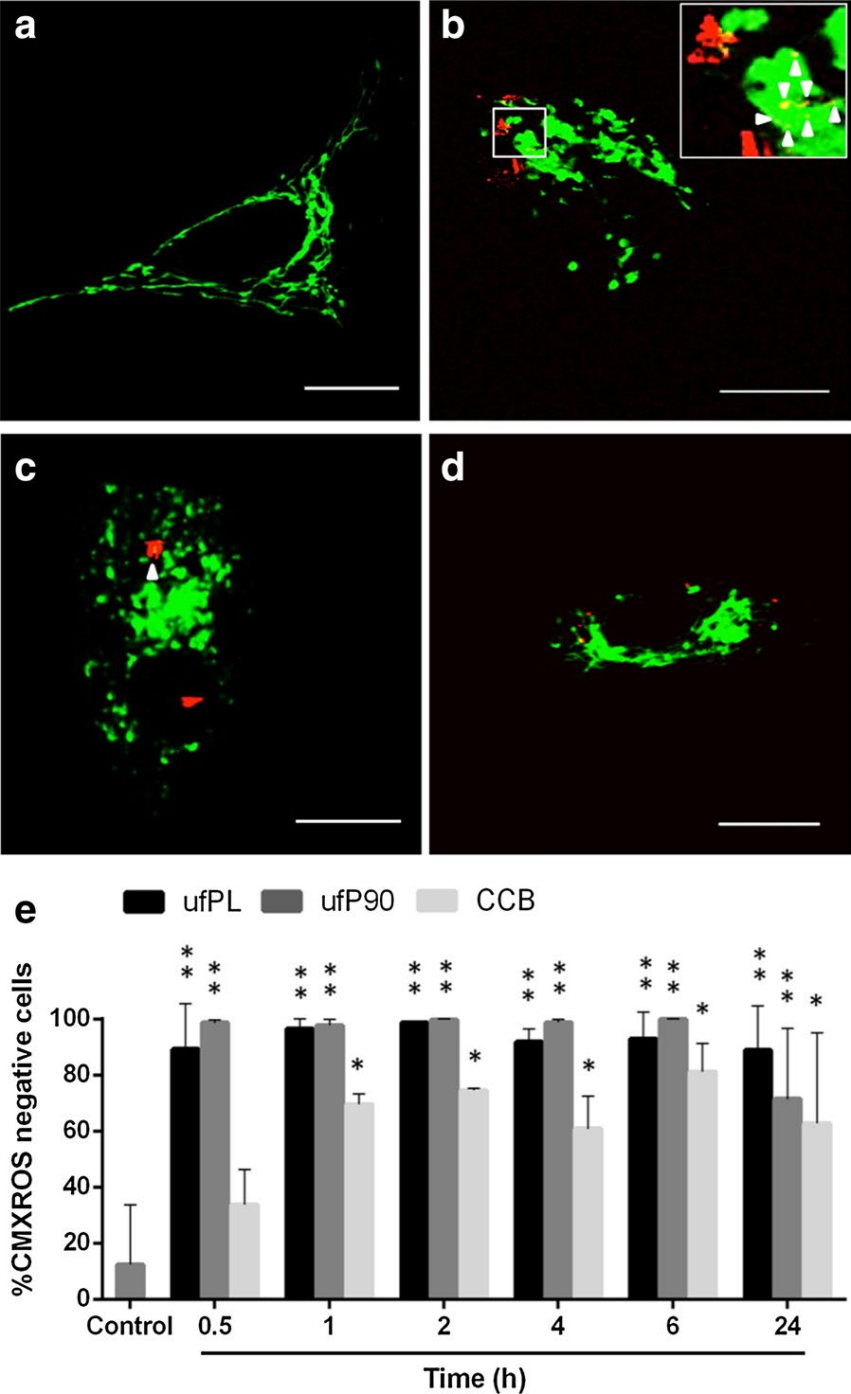
Mitochondrial damage by CBs in human lung fibroblasts. **a**–**d** Human lung fibroblasts (MRC-5 cell line) were exposed for 24 h to 20 µg/cm^2^ of three different types of CBs at 37 °C. Their mitochondrial organization was examined using CellLight^®^ Mitochondria-GFP (green, Ex/Em 488/510 nm, ~ 3 µW radiant power at the samples) and CB particles were imaged under femtosecond pulsed illumination (red, 4 mW average laser power at the samples, emission detection: 400–410 nm in non-descanned mode). Co-localization between CBs and mitochondria is yellow due to the overlapping colors and additionally indicated by arrowheads. Representative images are shown from: **a** control condition (0 µg/cm^2^), scale bar: 15 µm; **b** incubation with ufPL particles, scale bar: 5 µm; **c** incubation with ufP90 particles, scale bar: 5 µm; **d** incubation with CCB particles, scale bar: 10 µm. **e** Time course study (0.5–24 h) of the loss of mitochondrial membrane potential after exposure to 20 µg/cm^2^ of three different types of CBs at 37 °C. After incubation, the cells were labeled with CMXROS fluorochromes and the percentage of CMXROS negative cells was determined. Data are represented as means ± SD (n = 3). Statistically different from control marked by *(p < 0.05) and **(p < 0.0005)

First, the organization of the cellular mitochondria was evaluated using CellLight^®^ Mitochondria-GFP expressing fluorescent fusion proteins specifically targeting these organelles. The control cells (Fig. 4a) depicted the typical tubular-like organization indicating healthy mitochondria. In contrast, when the human lung fibroblasts were exposed to CBs (Fig. 4b–d) a substantial loss of the normal morphology of the mitochondrial organization occurred; they became more intermediate (tubular with swelling regions) and even fragmented (globular fragments).

Second, deposition of CBs in mitochondria was evaluated. The inset of Fig. 4b and the orthogonal projection from a z-stack throughout the cell depicted in Additional file 1: Figure S4A clearly show co-localization (indicated by arrowheads and/or yellow color) of the carbonaceous particles, visualized by probing their white-light generation as reported previously [13], and the mitochondria. The colocalization was further quantified by determining the Mander’s overlap coefficient (Additional file 1: Figure S4B). The ufPL particulates show an overlap with the mitochondria of 14 ± 11%. Also, the ufP90 particles show some co-localization with the mitochondrial organization but to a smaller extent (9 ± 8% Manders’ coefficient). In contrast, the larger, fine CCB particles do not show any co-localization with the cellular mitochondria.

Third, we examined the mitochondrial membrane potential (MMP) of the lung fibroblasts in a time course study using MitoTracker^®^ Red CMXROS (Fig. 4e). CMXROS is a fluorochrome that passively diffuses through the membrane of viable cells and is selectively sequestered in mitochondria with an active membrane potential. The study revealed a time-dependent decline of MMP when cells are exposed to CDPs compared to the control, which is significant within 30 or 60 min for the smallest and largest particles, respectively. In general, the maximum loss of MMP was reached after about 2 h of exposure. At this point, almost all cells incubated with ufPL and ufP90 lost their MMP, while 20% of the cells exposed to CCB remained unaffected.

### ATP production

**Fig. 5:**
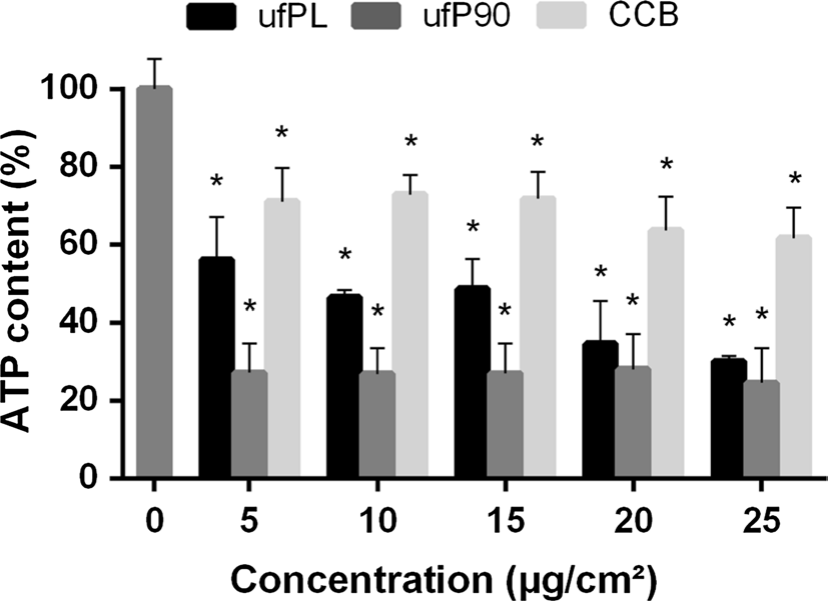
Intracellular ATP depletion by CBs in human lung fibroblasts. Human lung fibroblasts (MRC-5 cell line) were exposed to different concentrations (5–25 µg/cm^2^) of three different types of CBs for 24 h at 37 °C. Intracellular ATP content was determined by Cell-Titer glow luminescent cell viability assay. Data are represented as means ± SD (n = 3). * Statistically different from control p < 0.0001

The effect of CDP particles on the cellular adenosine triphosphate (ATP) level was evaluated employing a Cell-Titer glow luminescent cell viability assay. It is clear from the corresponding results (Fig. 5) that the metabolic activity—expressed by the depletion of ATP content—is affected significantly. Additionally, it is observed that the drop in the ATP level of cells treated with the ultrafine ufPL and ufP90 particles is more pronounced than fibroblasts incubated with the larger, fine CCB particulates.

### Antioxidants

To test if the observed toxicological effects are ROS-mediated, antioxidants were added to the culture medium of CDP exposed cells. Again, the metabolic activity expressed by the ATP content of the cells is measured after antioxidant treatment.

**Fig. 6:**
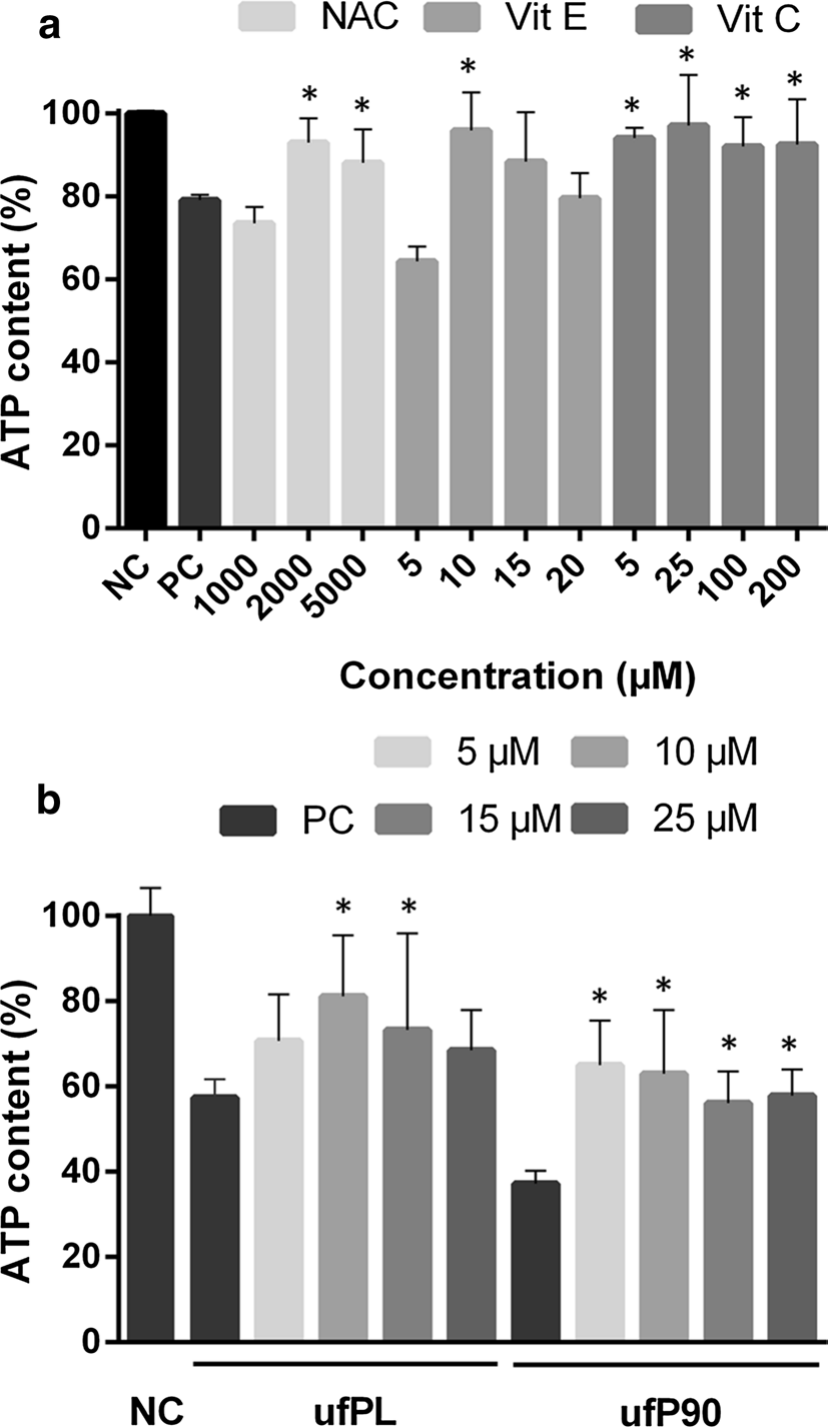
Improved ATP content in human lung fibroblasts exposed to CDPs and antioxidants. Human lung fibroblasts (MRC-5 cell line) were exposed to **a** fine particles or **b** ultrafine particles at a concentration of 20 µg/cm^2^ for 24 h at 37 °C. Next, **a** three different antioxidants (NAC, Vit. C and Vit. E for 2 h, 2 times 3 h and 24 h, respectively) or **b** Vit. C were added using the concentrations indicated in the graphs. Intracellular ATP content was determined by Cell-Titer glow luminescent cell viability assay. Positive control (PC), corresponding fine or ultrafine particles were added and negative control (NC) plain, complete culture medium was added. Data are represented as means ± SD (n = 5). * Statistically different from control p < 0.05

First, various concentrations of three different antioxidants, *N*-acetyl-l-cysteine (NAC), l-ascorbic acid (vitamin C) and α-tocopherol (vitamin E), were added to CCB exposed cells. The results show (Fig. 6a) that some specific concentrations of NAC and vitamin E significantly enhance the ATP content of the cells while vitamin C consistently increases the metabolic level from 79 ± 1% to 94 ± 3%.

Next, the concentration-dependent experiment was repeated wherein vitamin C, which showed the best and most consistent results in the previous experiment, was added to cells exposed to the ultrafine particulates under study. The corresponding results (Fig. 6b) indicate again a significant improvement of the ATP content of the cells from 55 ± 3% to 87 ± 9% for ufPL and 37 ± 3% to 65 ± 10% for ufP90.

Furthermore, the CDP exposed cells’ ability to remodel their surrounding matrix was tested after antioxidant treatment with vitamin C. The results depicted in Additional file 1: Figure S5 clearly display that under all conditions the collagen fibrils near the force poles of the cells are aligned and thus remodeled. No clear difference is visible anymore between the control cells and CDP exposed cells or between cells exposed to fine or ultrafine particles. This is in agreement with the results on the ATP content of the cells.

## Discussion

### CBs characterization

The CDP types employed in this study are commercial CB. CB is regularly synthesized and used in the global industry market, where employers are exposed to concentrations as high as 650 µg/cm^3^ [16]. In addition, the particles are representative for environmental CDPs to which humans are typically exposed and are often employed as a simplified model for soot or black carbon [17]. A major challenge is the use of relevant particle concentrations, since in vivo exposure cannot be converted directly to in vitro concentrations [18, 19]. To date, the majority of studies use unrealistically high particle concentrations despite the fact that a few studies have attempted to relate real-life particulate exposure to in vitro concentrations [18, 20]. These studies have shown that biologically relevant administered concentrations range between 0.2 and 20 µg/cm^2^. Yet, for occupational settings it is known that exposure concentrations can be much higher.

Before cellular exposure, the aggregation state of the CDP particles in aqueous environments was evaluated. All particle types tend to aggregate when suspended in ultrapure water or cell culture medium, which is clear from the TEM images [13] and measured hydrodynamic diameters on samples prepared from aqueous suspensions (Additional file 1: Table S1). This observation is in line with the literature showing similar particle aggregation in cell culture medium [21, 22]. Nevertheless, it has also been reported that aciniform aggregates can maintain the large surface area and other characteristics related to individual (ultra)fine particles [23, 24].

Endotoxins may have adverse effects that can mask the true biological effects of particles if their presence is overlooked [25]. While carbonaceous particles are naturally present in the atmosphere containing all sorts of contaminants, it is still important to have an idea about the possible contribution of endotoxins to observed toxicological effects. The performed LAL assay showed only for one of the three tested CDPs, CCB, a minimal endotoxin level. Note that this small endotoxin quantity is comparable and even lower than generally found in cell culture media and additives such as fetal bovine serum [26]. Additionally, the endotoxin level is lower than the current FDA limit (0.5 EU/mL) [27] and will not affect the cell culture studies [26].

### Cytoskeletal integrity

In a previous study, we have shown that CDP particles are capable of disturbing the tubulin cytoskeletal structure in human lung fibroblasts [13]. This observation warranted further in depth investigation of the actin cytoskeleton (Additional file 1: Figure S1). Also this cytoskeletal structure appeared to be affected by the presence of the CDP particles in a size-dependent way. Hence, we hypothesized that the cells become mechanically dysfunctional due to the cytoskeletal disturbances after CDP exposure, leading to impaired remodeling of the surrounding matrix.

## 3D cell-induced displacements and matrix remodeling

To test the hypothesis in a physiological relevant environment, human lung fibroblasts were embedded in a 3D collagen type I hydrogel. Under normal conditions, fibroblasts are able to remodel their surrounding matrix by attaching to collagen fibrils and contract them by exerting mechanical tension [28]. To date, collagen contraction assays are performed to evaluate matrix remodeling [29]. However, results from this type of assay lack crucial information such as changes in the interplay at ECM—cellular level and, moreover, the general result is biased by the proliferation rate of the embedded cells. Here, we studied cell-mediated matrix remodeling at the cellular level under normal and exposed conditions using 3D CDM [30]. As far as we know, CDM is employed for the first time to study toxicological effects of (nano)particles.

3D CDM turned out to be a powerful technique for the evaluation of the mechanical functioning of cells after particulate exposure. The method revealed at the individual cell-level that normal cell-induced displacements are heavily disturbed when cells are incubated with carbonaceous particles (Fig. 1). The label-free second harmonic imaging of the surrounding collagen matrix additionally strengthened this finding. The ECM remodeling around cells incubated with carbon-based particles is much less than the remodeling by control cells (Fig. 2). Moreover, both findings were size-dependent in which the smallest (ufPL and ufP90) particles inhibited the normal cell functioning and corresponding matrix remodeling more than their larger counterparts (CCB). The observed size-dependent effects are of significant relevance since especially the ultrafine particles can penetrate deep into the lung tissue where fibroblasts are residing. Moreover, it is certainly worrying that those smallest particulates have the ability to inhibit fibroblast-mediated matrix remodeling. It is in fact this cell type that has the responsibility to dynamically remodel the ECM during reparative processes in injured or diseased lungs.

A plausible explanation for these findings comprises impaired cell viability. Hence, the extent and mode of cell death was investigated (Additional file 1: Figure S3). Indeed, cells exposed to ultrafine but not fine particles show significant apoptotic death. Yet, inhibition of cellular displacements and matrix remodeling were also seen when cells are incubated with fine particles and moreover only local changes in the vicinity of viable (CellTracker positive) cells were analyzed. Hence, cell viability cannot explain the observed inhibition in matrix displacements and remodeling.

### ROS generation

Earlier literature reports have emphasized the pivotal role of oxidative stress in carbon-based particle toxicology [11, 31].

To check oxidative stress as a decisive toxicological factor, as a first step, the inherent oxidative ability of the different CBs in abiotic conditions was measured using a DTT assay.

Interestingly, all particles have similar oxidative potential (Fig. 3a). This is in contrast to literature where size-dependent effects are described [21, 32]. However, it should be noticed that only marginal differences of several nanomols in DTT consumption are reported when comparing different particle sizes and particle concentrations up to 100 µg/cm^2^ are used, which is fourfold higher than those employed in this study [32]. Additionally, the duration of the particles and DTT interaction is not always indicated. Here, we used the same procedure as for CB suspension preparation and cell experiments (for experimental details see “Methods” section), since this will give the most representative results.

Second, the role of oxidative stress under biotic conditions was studied and significant increase in cellular ROS production was observed, even at the lowest CB concentration (Fig. 3b). The vast majority of ROS is produced under biotic conditions, since at least eightfold ROS generation compared to the control is detected while only a maximum of 1.5-fold increase was found in abiotic condition. In agreement with earlier reports, no clear dose-dependent response curves are observed; the ROS production stagnates or even declines at higher doses [33, 34]. We hypothesize that the activation of a cellular antioxidant network, which counterbalances ROS at higher concentrations of CB, is responsible for this observation [35, 36].

Interestingly, while the particles did not exhibit differential inherent oxidative potential in the absence of cells, their intracellular ROS production significantly varies (p < 0.05). This difference in abiotic and biotic ROS production might be explained by the dissimilar amounts of internalized CBs (as observed by confocal microscopy, see Fig. 4a–d) or diverse interactions of the particulates with their biological target [21], which clearly indicates the need of multi-parametric analysis. Moreover, in line with the expectations, the analysis showed similar results for both ultrafine particles, while the ROS production of the fine particles was less.

### Interconnecting ROS production, mitochondrial damage and ATP depletion

Diverse (nano)particles varying in size and chemical composition have shown to induce structural damage and contribute to oxidative stress in mitochondria [22, 35]. Hence, the observed ROS production warrants further investigation of mitochondrial involvement in the toxicological outcome of CDPs.

First, the morphology and integrity of the mitochondria of the cells was evaluated. Mitochondria are morphologically dynamic organelles, which continuously undergo fission and fusion processes to form interconnecting tubular networks into small isolated organelles and vice versa [37]. This enables the cell to meet its metabolic needs and cope with internal or external stresses [38]. The control cells (Fig. 4a) showed the typical tubular morphology indicating healthy mitochondria. In contrast, substantial loss of the common morphology of the mitochondrial organization occurred when human lung fibroblasts were CDP exposed (Fig. 4b–d). The organelles became intermediate as in tubular with swelling regions and even fragmented [39]. This loss of the typical tubular morphology and resulting fragmentation is a strong demonstration of compromised mitochondrial dynamics. In fact, defects in the fission–fusion equilibrium will result in mitochondria that appear swollen and spherical, instead of tubular-like [40]. Overall, this outcome indicates that exposure to CDPs results in mitochondrial structural damage.

Deposition of CDPs in mitochondria may be the reason for mitochondrial damage. Li et al. [41] have already confirmed by employing electron microscopy that ultrafine particles preferentially localized in mitochondria, induce major structural damage and can contribute to oxidative stress. This is in agreement with our current findings. The microscopic images in Fig. 4 and Additional file 1: Figure S4 clearly show co-localization of the mitochondria with the ultrafine (ufPL and ufP90) but not fine (CCB) particles. This can explain the smaller extent of ROS production observed from the latter.

ROS production and subsequent oxidative stress may result in mitochondrial dysfunction. Mitochondria are the major sites of ROS production in mammalian cells [42]. During oxidative phosphorylation, oxygen is reduced to water through controlled addition of electrons via the respiratory chain. However, occasionally some of these electrons escape from the respiratory chain. Electron acceptance by molecular oxygen results in the formation of a range of ROS species such as superoxide anion radicals, hydrogen peroxides and hydroxyl radicals [43]. CDPs localized inside the mitochondria can alter their normal functioning and disrupt the electron transport chain due to either blocking electron transport or accepting an electron and transferring it to molecular oxygen [44]. The maintenance of the MMP in mitochondria is vital for proper oxidative phosphorylation functioning and is considered a critical marker to evaluate mitochondrial perturbation [45, 46]. In agreement with other studies, we found a time-dependent decline of MMP when cells are exposed to CDPs [47]. As loss in MMP alters normal functioning of the electron transport chain, this will ultimately result in enhanced ROS production in mitochondria leading to further mitochondrial membrane damage [45].

ATP is generated by oxidative phosphorylation in mitochondria [48]. As shown by the loss of MMP, damage is caused to the mitochondrial respiratory chain. Figure 5 clearly shows that the metabolic activity—expressed by the depletion of ATP content—is affected significantly when the cells are incubation with CDPs and this in a size-dependent manner. This is in agreement with the abovementioned results of mitochondrial damage.

Furthermore, the discussed interconnection between the ROS produced by the CDPs and the resulting cellular ATP depletion is additionally backed by the performed antioxidant experiments (Fig. 6). These experiments show that especially vitamin C can almost completely restore the ATP content in CCB and ufPL exposed cells and double the ATP level in ufP90 exposed cells. The latter is clearly indicative that the observed cellular toxicological effects are mainly driven by an oxidant-dependent mechanism elucidated by the CDPs. On the other hand, it also suggests that ROS scavenging, antioxidant treatments can restore these detrimental effects.

### ATP depletion as plausible inhibitory mechanism of cell-mediated matrix remodeling?

The key question that remains is how the CDP-induced toxicological effects relate to the impaired matrix displacements. We postulate that the carbon-induced metabolic arrest in the cells, expressed by ATP depletion, is a valid explanation for the disturbed cytoskeleton network and consequently the inhibited ECM remodeling. It is already known that ATP depletion in mammalian cells results in dramatic perturbation of the cytoskeleton. In 1980, Bershadsky et al. [49] already observed that inhibitors of the energy metabolism cause gradual disorganization of actin microfilament bundles in fibroblasts. Additionally, an almost identical mechanism of ATP-dependent cytoskeletal disruption after mitochondrial dysfunction in endothelial cells during simulated ischemia has been described [50]. Summarized, ATP is crucial for the polymerization of the actin component of the cytoskeleton and is therefore required to maintain its structural integrity [51]. The actin network regulates cell shape and the distribution of stresses on the substrate, thereby mediating the mechanical interactions of the cell with the ECM [52–54]. Consequently, if the network is disturbed this may lead to impaired force generation. Note, while this proposed mechanism seems to be contradicted by the inverse difference between the smallest particles (ufPL and ufP90) seen for the ROS and ATP assays on the one hand and cellular displacements on the other hand, only significant differences (p < 0.05) between both particles could be observed for the ROS assay. Furthermore, there is a strong correlation (Pearson correlation coefficient = 0.87; Goodness of fit R^2^ = 0.76) between the observations made for mitochondrial dysfunction and the impaired matrix displacements strengthening our postulated hypothesis. Even stronger evidence includes the imaging of the collagen fibril organization remodeled by CDP exposed cells after antioxidant treatment (Additional file 1: Figure S5). While CDP exposed cells lose their ability to remodel their surrounding matrix, they regain their remodeling potential after antioxidant treatment. Hence, the restoration of the cellular ATP level is reflected in recovery of the matrix remodeling by those cells. In summary, the proposed toxicological mechanism of action of the CDPs in the MRC-5 cells is summarized in Additional file 1: Figure S6.

## Conclusions

Due to the inevitable exposure of humans to polluting combustion-derived particles it is imperative to investigate their potential detrimental effects on human lung cells. In the present study we have proven that carbonaceous particles induce various deleterious effects via an oxidant-dependent mechanism, including: (i) ROS production, (ii) mitochondrial damage and dysfunction, and (iii) ATP depletion. Furthermore, it was shown that matrix displacements were disturbed when human lung fibroblasts were exposed to CB particles, resulting in the inhibition of matrix remodeling by these cells. Since ATP is a key component for cell-induced matrix contraction, the described oxidant-dependent toxicological mechanism can be a valid explanation for the observed impaired cell-induced remodeling. Moreover, it can be concluded that ultrafine particles cause more adverse effects in human lung fibroblasts compared to their larger counterparts.

These novel insights are of critical importance, since they show that ultrafine carbonaceous particles have the ability to inhibit the physiological relevant function of fibroblasts, namely their mechanical functionality to remodel the extracellular matrix. Dysfunctional fibroblasts may eventually lead to loss of lung tissue structure and functionality. This provides essential information on how those nanoparticles are destructively involved in e.g. impaired tissue remodeling or repair processes in lung damage and diseases associated to inhalation of particulate matter. It also suggests new potential strategies to attenuate the toxicity induced by carbonaceous particle exposure, like for example ROS scavenging antioxidant treatment.

## Methods

### Characterization of CB particles

Three types of carbon black particles (CBs) were tested: ultrafine (uf) carbon black nanopowder (ufPL; PlasmaChem GmbH, Germany), ultrafine Printex 90 (ufP90; Orion Engineered Carbons, Germany) and conductive carbon black nanopowder (CCB; US Research Nanomaterials, USA). Detailed particle characterization was performed in our previous study [13]. For the convenience of the reader, we summarized the main characteristics in Additional file 1: Table S1.

### Cell culture

Human lung fibroblasts (MRC-5 cell line, ATCC CCL-171, LGC Standards, France) were cultured (37 °C, 5% CO_2_) in Minimum Essential Medium (MEM) supplemented with 10% fetal bovine serum (FBS; Biochrom AG, Germany), 100 U/mL penicillin, and 100 µg/mL streptomycin.

### Confocal imaging

All imaging was performed using a Zeiss LSM510 META NLO scan head mounted on an inverted laser-scanning microscope (Zeiss Axiovert 200 M; Zeiss, Germany) equipped with a 40×/1.1 water immersion objective.

### Cell-induced matrix displacements imaging and quantification

The effect of CB particles on the displacements generated by cells was evaluated using cell-induced displacement microscopy according to a method described by us previously [30].

### Collagen type I matrix remodeling imaging

Second harmonic imaging of the fibrils of contracted hydrogels and acquisition of the cell body was done as in the Additional file 1.

### General toxicological assays

The materials and methods of the following assays can be found in the Additional file 1: cell death analysis, abiotic ROS generation, biotic ROS generation, mitochondrial organization and colocalization imaging, mitochondrial functioning assay, metabolic activity assay and antioxidant treatment testing.

### Statistical analysis

In general, every experiment was repeated three times with triplicates of each condition. Data are represented as mean ± standard deviation and were analyzed using the commercially available software GRAPHPAD (Graphpad Prism 6, Graphpad Software Inc., USA) and JMP (JMP Pro 12, SAS Institute Inc., USA). Analysis of variance (ANOVA) or linear mixed model followed by the post-test Dunnett, for multiple comparisons, were performed.

## Additional file

**Additional file 1.** Detailed materials and methods and additional figures.

## Abbreviations

2D: two-dimensional
3D: three-dimensional
ANOVA: analysis of variance
ATP: adenosine triphosphate
BC: black carbon
BSA: bovine serum albumin
CB: carbon black
CCB: conductive carbon black particles
CDPs: combustion-derived particles
DCF-DA: 2′,7′-dichlorodihydrofluorescein diacetate
DTT: dithiothreitol
ECM: extracellular matrix
EU: endotoxin units
FBS: fetal bovine serum
fs: femtosecond
IMDM: Iscove’s modified Dulbecco’s medium
LAL: limulus amebocyte lysate
MEM: minimum essential medium
MRC-5: human lung fibroblast cell line
NC: negative control
PBS: phosphate buffered saline
ROS: reactive oxygen species
SD: standard deviation
SHG: second harmonic generation
TEM: transmission electron microscopy
ufP90: ultrafine carbon black (Printex 90) particles from Orion Engineered Carbons
ufPL: ultrafine carbon black particles from PlasmaChem
