# Supplementary material for: Combustion-derived particles inhibit in vitro human lung fibroblast-mediated matrix remodeling
Source: J Nanobiotechnology. 2018 Oct 27;16:82. doi: 10.1186/s12951-018-0410-x (PMC6204012; doi:10.1186/s12951-018-0410-x)
Supplement: Supplementary file 1 — Additional file 1. Detailed materials and methods and additional figures. [file 12951_2018_410_MOESM1_ESM.docx]

# Additional file 1

Combustion-Derived Particles Inhibit *in vitro* Human Lung Fibroblast-Mediated Matrix Remodeling

*Hannelore Bové^1,2*^, Jens Devoght^1^, Leentje Rasking^1^, Martijn Peters^3^, Eli Slenders^1^, Maarten Roeffaers^2^, Alvaro Jorge-Peñas^4^, Hans Van Oosterwyck^4,5^, Marcel Ameloot^1^*

^1^ Biomedical Research Institute, Hasselt University, Agoralaan Building C, Diepenbeek, Belgium; ^2^ Centre for Surface Chemistry and Catalysis, KU Leuven, Celestijnenlaan 200F, Louvain, Belgium; ^3^ Institute for Materials Research, Hasselt University, Agoralaan Building D, Diepenbeek, Belgium; ^4^ Department of Mechanical Engineering, KU Leuven, Celestijnenlaan 300C – box 2419, Louvain, Belgium; ^5^ Prometheus, div. Skeletal Tissue Engineering, KU Leuven, Louvain, Belgium

[hannelore.bove@uhasselt.be](mailto:Hannelore.bove@uhasselt.be) (*corresponding author), [jens.devoght@uhasselt.be](mailto:jens.devoght@uhasselt.be), [leentje.rasking@student.uhasselt.be](mailto:leentje.rasking@student.uhasselt.be), [martijn.peters@uhasselt.be](mailto:martijn.peters@uhasselt.be), [eli.slenders@uhasselt.be](mailto:eli.slenders@uhasselt.be), [maarten.roeffaers@kuleuven.be](mailto:maarten.roeffaers@kuleuven.be), [Alvaro.JorgePenas@kuleuven.be](mailto:Alvaro.JorgePenas@kuleuven.be), [Hans.VanOosterwyck@kuleuven.be](mailto:Hans.VanOosterwyck@kuleuven.be), [marcel.ameloot@uhasselt.be](mailto:marcel.ameloot@uhasselt.be).

MATERIALS AND METHODS

# *Characterization of CB particles*

Endotoxin concentrations were quantified with the Pierce Limulus Amebocyte Lysate (LAL) chromogenic endotoxin quantitation kit (Fisher Scientific, Belgium). The assay was performed according to the manufacturer’s instructions. Particles in Iscove’s Modified Dulbecco’s Medium (IMDM; Life Technologies, Belgium) were sonicated as described below. The particle suspensions were centrifuged for 30 min at 22,000 g and the supernatants were collected and centrifuged using the same protocol. This procedure of supernatant centrifugation was repeated three times and the endotoxin levels in the resulting supernatants were determined. Four endotoxin standards (0.1 to 1 EU/mL) were used to generate a standard curve to calculate endotoxin levels. The light absorbance was monitored at 410 nm using the microplate reader Fluostar Optima (BMG Labtech, Belgium).

***Carbon black stock suspensions***

Stock suspensions of CBs (2 mg/mL) were prepared in IMDM supplemented with 10% fetal bovine serum (FBS; Biochrom AG, Germany) and 1 % penicillin streptomycin by sonicating 30 min using an ultrasonic bath (Branson 5800, 40 kHz, Emerson, USA) and stored at 4°C in the dark until further use. Prior to utilization, stock suspensions were sonicated in a water bath for 20 min. Working suspensions were prepared in complete IMDM immediately before experiments.

***CB treatment of cells***

Just before the experiments, CB stock solutions were sonicated for 20 min and diluted to required concentrations using complete IMDM. Cells were exposed to CB concentrations of 5, 10, 15, 20, and 25 *µ*g/cm² for generally 24 h. To expose cells to identical CB concentration, the doses were adapted considering the corresponding cultured surface and volumes.

# *Actin cytoskeleton imaging*

Cells exposed to vehicle or CB solutions were fixed with 4 % paraformaldehyde containing 4 % sucrose in PBS for 20 min. Permeabilization was performed using 0.3 % Triton X-100 for 30 min followed by a 1 h block with 2 % BSA in PBS. The actin staining solution of phalloidin Alexa Fluor 647 (Life Technologies, Belgium) diluted 1:40 in PBS containing 2 % BSA and 0.1 % Triton X-100 was incubated for 1 h. Between different experimental steps, the cells were washed three times with PBS. Before confocal imaging, all wells were aspired and 200 *µ*L Immu-Mount (Thermo Scientific Shandon^TM^ Immu-Mount^TM^, Thermo Fisher Scientific) was added. Images of the stained cells were acquired using the confocal system as described below.

For imaging the actin cytoskeleton, excitation at 633 nm was performed using a 5 mW Helium Neon laser (LASOS Lasertechnik GmbH, Germany, 3 *μ*W maximum radiant power at the sample). Band-pass filter 650 – 710 nm was used for filtering the emission signal. The resulting 1024x1024 images with a pixel size of 0.17 *μ*m were recorded with a pixel dwell time of 9.6 *μ*s. Images were captured using the Zeiss AIM 4.2 software and processed with the image-processing program Fiji (ImageJ v1.47, open source software, <http://fiji.sc/Fiji>).

***Cell-induced matrix displacements imaging and quantification***

*Collagen gel preparation and contraction*

Briefly, cells were seeded at a density of 15,000 cells/well in a 8-well Ibidi culture plate (Ibidi GmbH, Planegg, Germany) and incubated with vehicle (complete culture medium) or three different types of CB particles at 20 µg/cm^2^ for 24 h. Next, the cells were washed three times with PBS and stained for 45 min with 12.5 µM CellTracker^TM^ Green CMFDA (Life Technologies) in serum-free IMDM. The CellTracker^TM^ stain allows the discrimination of the vital from the dead cells during the CDM measurements. Next, cells were washed three times with PBS, detached and embedded in a 3D collagen type I hydrogel at a final concentration of 15,000 cells/mL collagen.

Hydrogels were prepared on ice by mixing 8 volumes of collagen consisting of 1:2 ratio of rat tail collagen (collagen type I, 10.31 mg/mL, Corning, The Netherlands) and bovine skin collagen (collagen type I, 5.9 mg/mL, Nutragen, Advanced Biomatrix, Germany) in complete MEM at a final concentration of 2.4 mg/mL diluted with an appropriate amount of 10x MEM. Next, 10% (vol/vol) sodium bicarbonate (23 mg/mL) containing 1.5 mg/mL fluorescent polystyrene beads (0.2 *μ*m diameter, carboxylated, ex/em 580/605, Invitrogen, Belgium) was added. The pH of the mixture was neutralized using 1 M sodium hydroxide, after which 1 volume of cells were embedded.

*Displacements imaging*

The hydrogels including the exposed cells were allowed to set for 18 h at 37°C before the start of the displacement experiments. Imaging was performed using the confocal microscopy system as described above. The second harmonic signal from the collagen fibrils was acquired employing a femtosecond pulsed laser (~ 4 mW average laser power at the sample, 810 nm, 150 fs, 80 MHz, MaiTai DeepSee, Spectra-Physics, USA) as excitation source at an average laser power of 5 mW measured on the stage and the emission signals were epi-collected by an analogue photomultiplier tube (Zeiss) after passing a 442 nm dichroic beam splitter and a 5 nm wide band pass filter with a central wavelength of 405 nm. For imaging the CellTracker^TM^-labeled cells inside the hydrogels, a 30 mW air-cooled Argon ion laser (LASOS Lasertechnik GmbH, Germany) emitting at 488 nm (~ 3 μW maximum radiant power at the sample) was employed. A band-pass filter 500 – 530 nm was used for filtering the emission signal.

An image stack of a collagen volume (173 x 173 x 45 *µ*m^3^ on average) around the cell of interest was acquired at 37°C and 5% CO_2_ by means of a stage incubator (Tempcontrol 37-2 digital, PeCon, Erbach, Germany). After imaging the hydrogel under cellular tractions, the embedded cells were treated with 25 *µ*M cytochalasin B and re-imaged until a force-free relaxed state of the hydrogel was achieved.

*Displacements quantification*

Images were captured using the AIM 4.2 software. Calculation of the displacement fields was performed from the fibril images using non-rigid image registration as described previously by our research groups.(Jorge-Penas et al. 2017)

***Collagen type I matrix remodeling imaging and quantification***

*Remodeling quantification*

Local changes in cell-mediated collagen type I matrix remodeling were analyzed at the axial zone near the cellular force poles. At each force pole, a zone of 50 x 50 µm² was cropped and the collagen fibrils were traced manually with the image-processing program Fiji. For each cropped image, the distribution of fibril orientations was analyzed using the Von Mises distribution given by equation 1:

$\Phi\left( \theta\right)\propto{A+B e}^{\kappa cos2(\theta-\mu)}$ (1)

This analysis yields the spread of fibril orientation *κ*, which was used to compare the collagen matrix remodeling capacity by the cells exposed to various CDP types. Note, a *κ* value near zero points to a large spread of the angle (*θ*) distribution for the collagen fibrils around the average value (*μ*), while larger *κ* values indicates a narrow distribution and thus preferential direction of fibril organization inside the hydrogel.

***Cell death analysis***

To assess the extent and mode of cell death, Annexin V and propidium iodide labeling was carried out. After vehicle or CB treatment, cells were collected and washed twice with cold cell staining buffer (BioLegend, United Kingdom). The cells were resuspended in Annexin V binding buffer (BioLegend) at a concentration of 10^6^ cells/mL and stained using 4.55 µg/mL FITC Annexin V (BioLegend) and 23 µg/mL propidium iodide (Sigma Aldrich). The staining solution was incubated in the dark for 15 min at room temperature after which the cells were analyzed by flow cytometry (BD Fortessa flow cytometer; BD Biosciences, Belgium).

# *Detection of abiotic and biotic ROS generation*

Intrinsic ability of CB particles to generate reactive oxygen species (ROS) in abiotic conditions was measured by a dithiothreitol assay (DTT; Fisher Scientific) as described by Koike *et al.* (Koike and Kobayashi 2006). In short, the oxidation of 50 *µ*L of 1 mM DTT in Tris-HCl buffer (1 mM, pH 8.0) by ROS generated by CB solutions (5 – 25 *µ*g/cm², prepared as described above) was determined by 5-minute incubation with 50 *µ*L of 5’5-dithiobis(2-nitrobenzoic acid) (0.1 mM in 0.1 M Tris-HCl buffer pH 8.0, DTNB; Fisher Scientific). The formation of 5-mercapto-2-nitrobenzoic acid (TNB) was measured by a Fluostar Optima spectrophotometer at 412 nm. Blanks and samples were run in triplicate.

Biotic ROS generation was monitored by employing a 2’,7’-dichlorodihydrofluorescein diacetate (DCF-DA, Sigma Aldrich) staining. Dose-dependent measurements were performed by incubating cells with CB particles (5 – 25 µg/cm², 24 h), followed by staining with 20 µM DCF-DA for 15 min at 37 °C. Cells were washed three times with PBS and analyzed using a BD Fortessa flow cytometer at excitation and emission wavelengths of 488 and 530 nm, respectively. For each sample, an analysis of 10,000 cells was performed.

# *Mitochondrial organization and colocalization imaging*

Mitochondrial organization of control and CB-treated (20 µg/cm² of ufPL, ufP90 and CCB; 24 h) cells was evaluated by staining the mitochondria overnight (16 h) with 2 µL (= 200,000 particles) CellLight^®^ Mitochondria-GFP, BacMam 2.0 (Thermo Fisher Scientific, Germany) in 350 µL culture medium per well.

Images of the stained mitochondria were acquired using the microscope set-up as described above. A 30 mW air-cooled Argon ion laser (LASOS Lasertechnik GmbH, Germany) emitting at 488 nm (~ 3 *μ*W maximum radiant power at the sample) was used as excitation source and a band-pass filter 500 – 530 nm was used for filtering the emission light. CB particles were visualized by femtosecond pulsed laser excitation at 810 nm and filtering of the emission signal by a 400 – 410 nm band-pass filter in the non-descanned mode. The resulting 1024x1024 images with a pixel size of 0.09 *μ*m were recorded with a pixel dwell time of 12.8 *μ*s. Images were captured using the AIM 4.2 software and processed with the image-processing program Fiji.

Colocalization analysis of CDPs with mitochondria was performed by calculating the Manders’ overlap coefficient using the JACoP plug-in in Fiji. Prior to analysis, a threshold was set to the estimated background value. The colocalization coefficient was defined as the fraction of CDPs overlapping with mitochondria. Obtained coefficients are not dependent on the relative intensities of each channel and cross-talk between the channels was found to be negligible.

# *Mitochondrial functioning assay*

Mitochondrial membrane potential dynamics were studied using the mitochondrial selective probe MitoTracker^®^ Red (CMXROS; Life Technologies) according to manufacturer’s recommendations. In short, cells were prepared as described above and exposed to 20 µg/cm² of CB suspensions for various time periods (0.5, 1, 2, 4, 6, 24 h). Following three washing steps, cells were incubated with 500 nM CMXROS for 15 min. Analysis of 10,000 cells was performed on a BD Fortessa flow cytometer using 488 nm excitation and 615 nm emission wavelengths.

***Metabolic activity assay and antioxidant treatments***

Metabolic activity of CB-treated cells was measured using Cell-Titer Glo luminescent cell viability assay (Promega, The Netherlands) according to supplier’s instructions. Cells were cultured as described above, but in white opaque walled 96-well plates (Greiner Bio-One, Belgium). Luminescence was recorded by a Fluostar Optima spectrophotometer and controls were included to correct for autoluminescence.

# To study the effect of antioxidants on cells treated with CDPs, three different antioxidant agents were added to the culture medium: N-acetyl-L-cysteine (NAC; Sigma Aldrich), L-ascorbic acid (Vitamin C; Sigma Aldrich) and α-tocopherol (Vitamin E; Sigma Aldrich). The employed incubation times were 2 h, 2 times 3 h and 24 h, respectively. The concentrations are indicated on the corresponding graphs.

Table S1: Physicochemical characteristics of the three types of carbon black particles used.


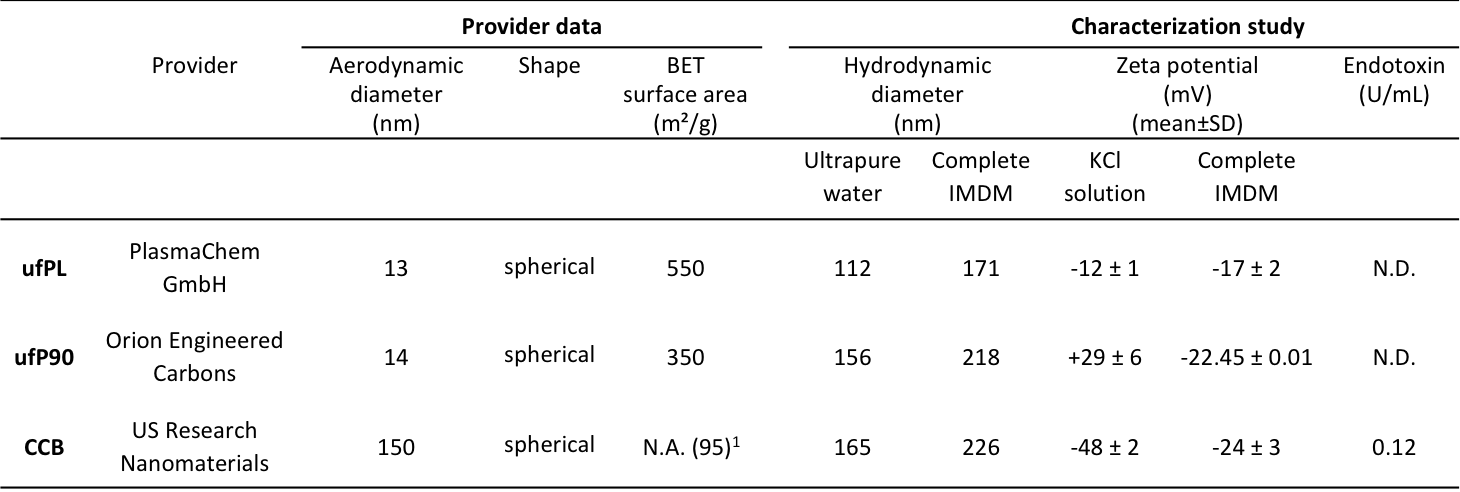


^1^The value was estimated according to (Thiele et al. 2010) N.A.: not applicable. BET: Brunauer, Emmett and Teller; specific surface area of a sample. N.D: not detectable.


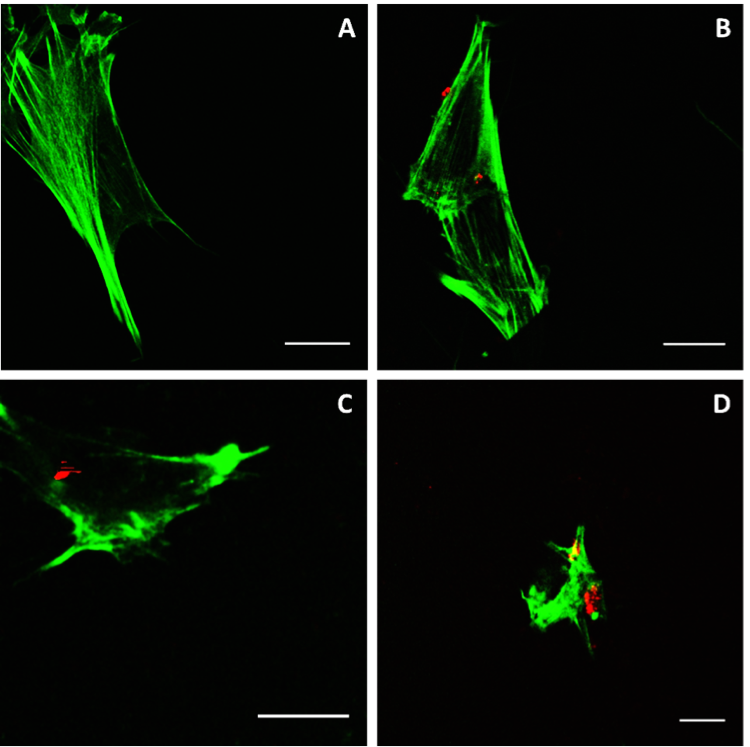


**Figure S1:** Disturbance of the actin cytoskeleton of MRC-5 lung fibroblasts by engulfed carbon particles. Actin cytoskeleton (green, Ex/Em 650/668 nm, ~ 3 *µ*W radiant power at the sample) of normal human lung fibroblasts incubated with 20 *µ*g/cm^2^ ufPL particles (red, 4 mW average laser power at the sample, emission detection: 400 – 410 nm in non-descanned mode) at 37 °C. (A) Control cells. (B) 4 h incubation. (C) 8 h incubation. (D) 24 h incubation. Scale bars: 20 *µ*m.


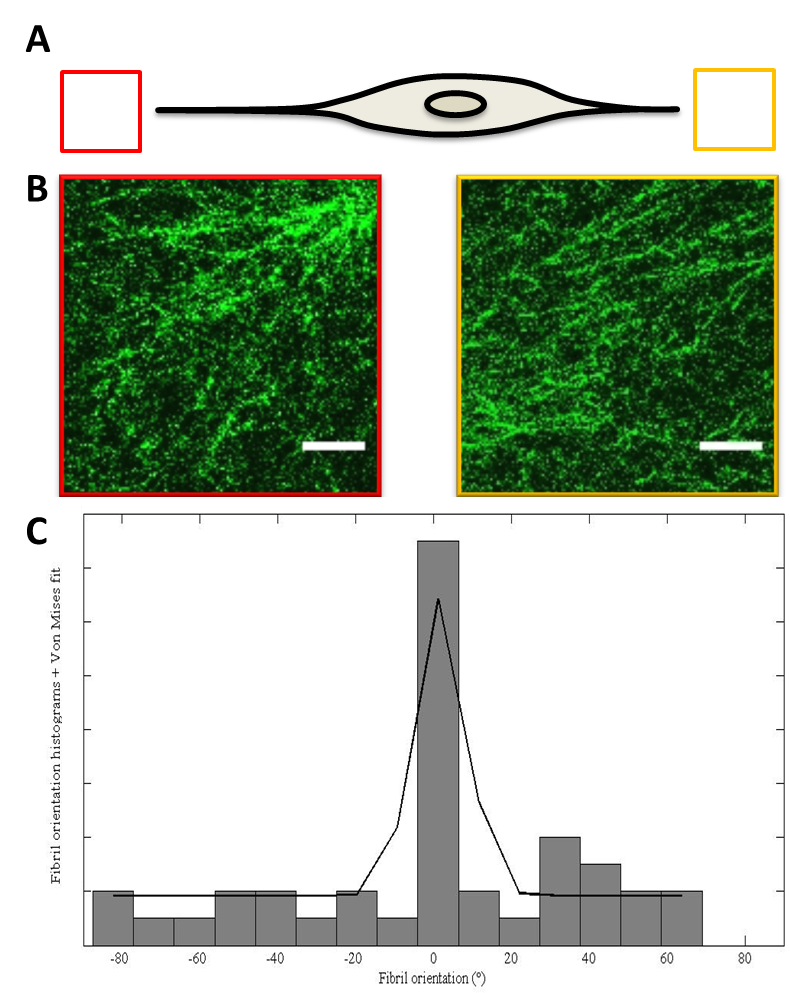


Figure S2: Detection of local changes in cell-mediated collagen hydrogel remodeling by quantification of the fibril orientation. (A) Schematic representation of the selected axial zones near the cellular force poles. (B) Both axial 50 x 50 *µ*m² areas of the second harmonic generation image of the collagen fibrils were cropped for the analysis. Scale bars: 10 *µ*m. (C) Example histogram with fitted Von Mises function of manually determined and normalized fibril orientations of a collagen hydrogel containing control cells.


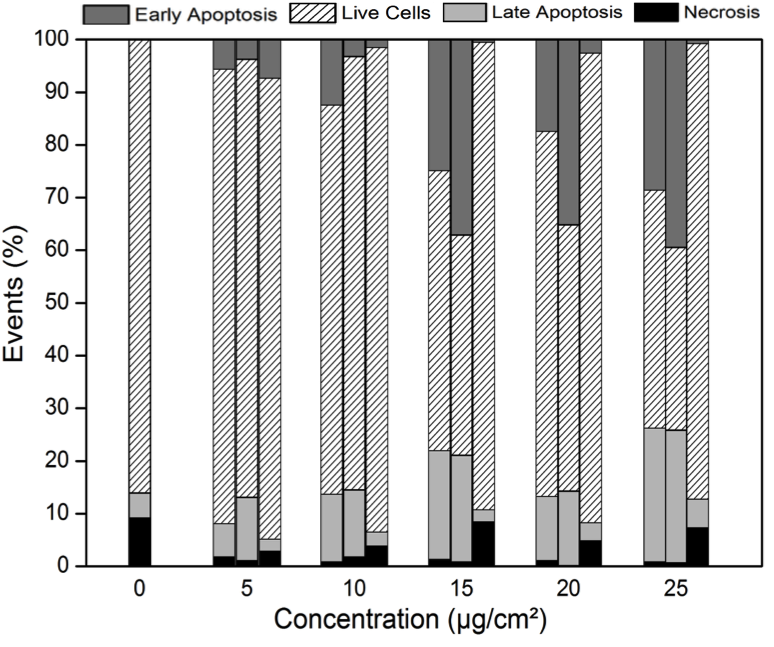


Figure S3: Extent and mode of cell death of human lung fibroblasts after CB exposure. Human lung fibroblasts (MRC-5 cell line) were exposed to different concentrations (5 – 25 µg/cm²) of three different types of CBs for 24 hours at 37°C. For each concentration, from left to right: ufPL, ufP90 and CCB. At the end of the exposure an Annexin V – propidium iodide assay was conducted to determine the extent and mode (live, early or late apoptosis and necrosis) of cell death.


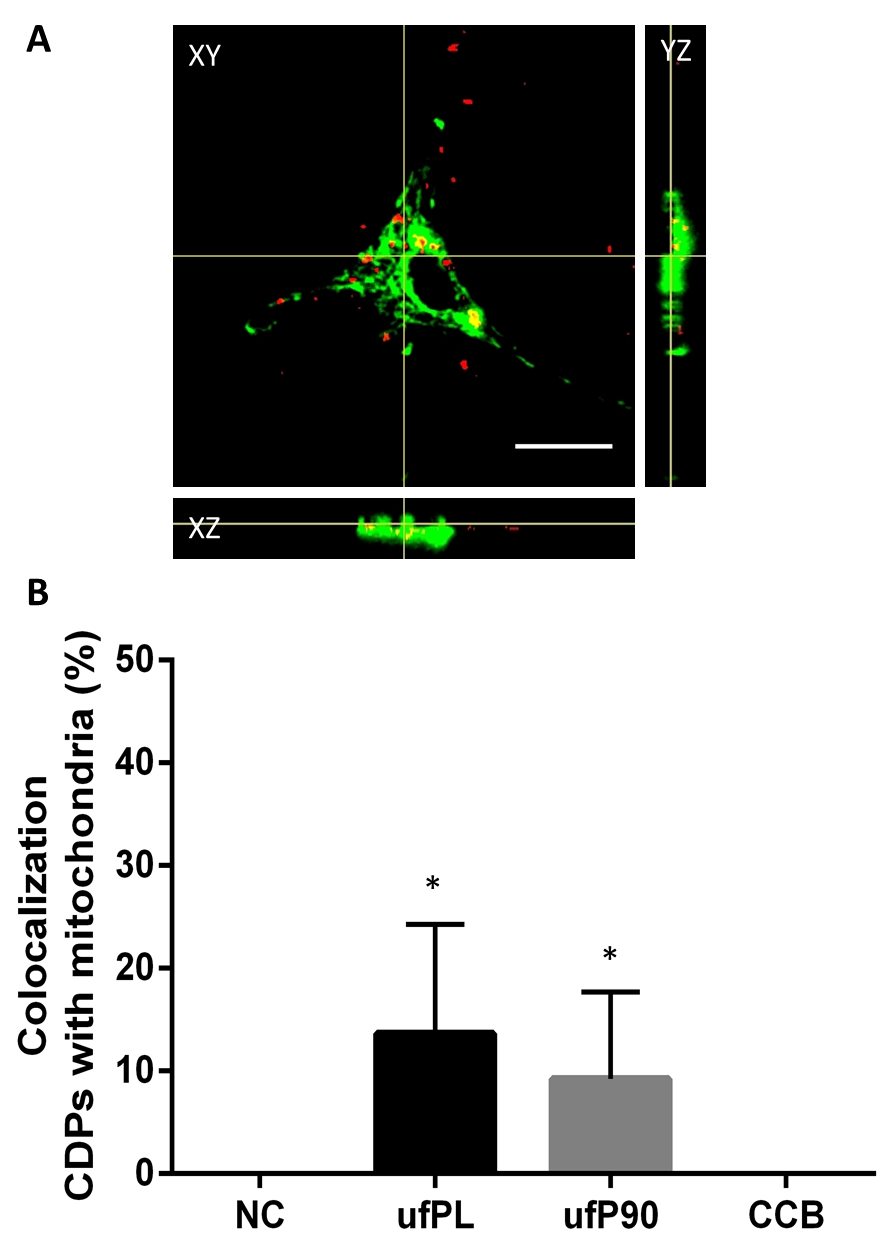


Figure S4: Colocalization of CDP with cellular mitochondria. Human lung fibroblasts (MRC-5 cell line) were exposed for 24 hours to 20 µg/cm² of three different types of CBs at 37°C. (A) The mitochondria were examined using CellLight^®^ Mitochondria-GFP (green, Ex/Em 488/510 nm, ~3 *µ*W radiant power at the samples) and the CDPs were imaged under femtosecond pulsed illumination (red, 4 mW average laser power at the samples, emission detection: 400 – 410 nm in non-descanned mode). Colocalization between CDPs and mitochondria is yellow due to the overlapping colors. To show CDP colocalization with mitochondria, an orthogonal view of a z-stack throughout the cell is shown. Scale bar: 30 *µ*m. (B) Colocalization is quantified using the Manders’ coefficient. Data are represented as means±SD (n = 40 cells/condition). Statistically different from control marked by * (p < 0.0001).


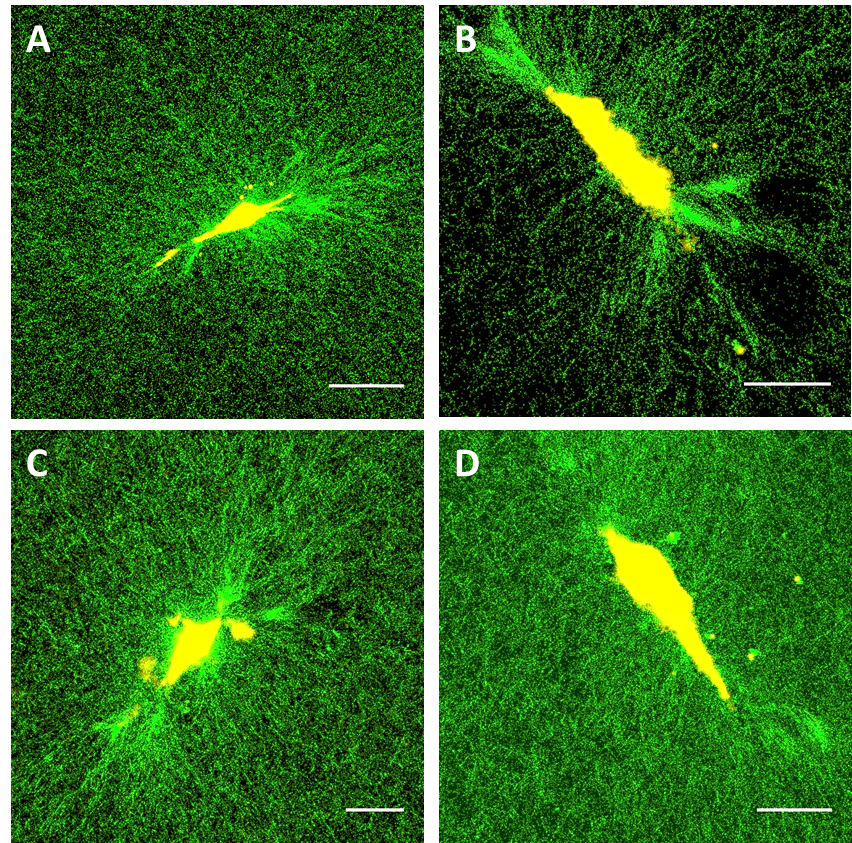


Figure S5: Restored collagen type I matrix remodeling by human lung fibroblasts exposed to CDPs after antioxidant treatment with L-ascorbic acid. Human lung fibroblasts (MRC-5 cell line) were exposed for 24 hours to 20 *µ*g/cm² of three different types of CDPs at 37°C and afterwards treated with L-ascorbic acid (2 times 3 h). Next, the cells are embedded in 3D collagen type I hydrogels to study cell-mediated matrix remodeling. Representative images of collagen type I (second harmonic imaging, green) remodeling induced by fibroblasts (cell body in yellow) are shown incubated with (A) culture medium (negative control), (B) ufPL, (C) ufP90 and (D) CCB. Scale bars: 30 *µ*m.


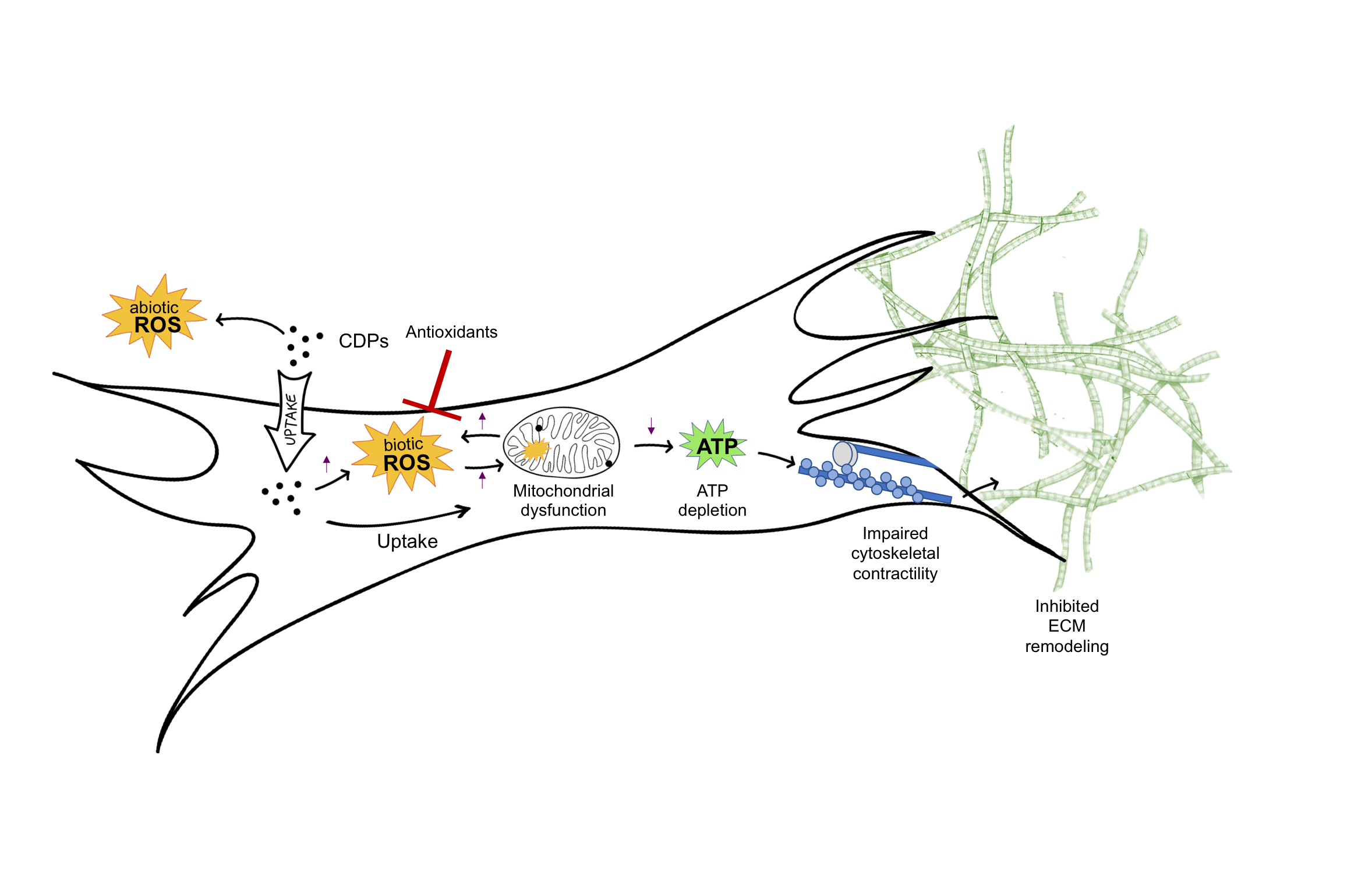


**Figure S6.** Schematic figure summarizing the toxicological mechanism of action of the CDPs in human lung fibroblasts (MRC-5 cell line).

**References**

Jorge-Penas A, Bové H, Sanen K, et al. (2017) 3D full-field quantification of cell-induced large deformation in fibrillary biomaterials by combining non-rigid image registration with label-free second harmonic generation. Biomaterials 136:86-97

Koike E, Kobayashi T (2006) Chemical and biological oxidative effects of carbon black nanoparticles. Chemosphere 65(6):946-951

Thiele G, Poston M, Brown R (2010) A case study in sizing nanoparticles. In: Micromeritics Analytical Services and MVA Scientific Consultants. <http://www.micromeritics.com/Repository/Files/A_Case_study_in_Sizing_Nano_Particles.pdf> Accessed [15/05/2017
